# Thermal Surface Properties, London Dispersive and Polar Surface Energy of Graphene and Carbon Materials Using Inverse Gas Chromatography at Infinite Dilution

**DOI:** 10.3390/molecules29122871

**Published:** 2024-06-17

**Authors:** Tayssir Hamieh

**Affiliations:** 1 Faculty of Science and Engineering, Maastricht University, P.O. Box 616, 6200 MD Maastricht, The Netherlands; t.hamieh@maastrichtuniversity.nl; 2 Laboratory of Materials, Catalysis, Environment and Analytical Methods (MCEMA), Faculty of Sciences, Lebanese University, Beirut P.O. Box 6573/14, Lebanon

**Keywords:** London dispersion equation, Hamieh thermal model, thermal conductivity, London dispersive and polar surface energy, Lewis acid–base constants, coupling amphoteric constant, average separation distance between particles, acid and base surface energies

## Abstract

The thermal surface properties of graphenes and carbon materials are of crucial importance in the chemistry of materials, chemical engineering, and many industrial processes. Background: The determination of these surface properties is carried out using inverse gas chromatography at infinite dilution, which leads to the retention volume of organic solvents adsorbed on solid surfaces. This experimental and fundamental parameter actually reflects the surface thermodynamic interactions between injected probes and solid substrates. Methods: The London dispersion equation and the Hamieh thermal model are used to quantify the London dispersive and polar surface energy of graphenes and carbon fibers as well their Lewis acid-base constants by introducing the coupling amphoteric constant of materials. Results: The London dispersive and polar acid-base surface energies, the free energy of adsorption, the polar enthalpy and entropy, and the Lewis acid-base constants of graphenes and carbon materials are determined. Conclusions: It is shown that graphene exhibited the highest values of London dispersive surface energy, polar surface energy, and Lewis acid-base constants. The highest characteristics of graphene justify its great potentiality and uses in many industrial applications.

## 1. Introduction

The determination of the thermal surface properties of materials and their dispersive, polar, and Lewis acid-base energies is of great interest in many industrial applications and fundamental sciences. These various surface properties are directly correlated with the interactions between materials and adsorbents and play an important role in several scientific disciplines such as adhesion, adsorption, diffusion, evaporation, condensation, coatings, friction, conduction, chemical engineering, catalysis, and electronics. The behavior of materials strongly depends on their surface properties and the dependency of such properties on the temperature. Indeed, the temperature exerts an important effect on the interactions between particles or molecules, their London dispersive and polar surface energies, and their Lewis acid-base properties. The variations against the temperature in the adhesive, two-dimensional state, and surface properties of materials such as oxides, polymers, composites, graphite, and carbon are generally used in many industrial processes.

Carbon materials, forming a large variety of allotropes, present very interesting physicochemical and mechanical properties, being used to replace conventional metals for various applications by decreasing the weight of products, and they are quite famous for their thermal properties. Indeed, the thermal conductivity of carbon allotropes varies from 0.01 W m^−1^ K^−1^ in amorphous carbon to more than 2000 W m^−1^ K^−1^ in diamond or graphene [1].

Graphene exfoliation [2] led to the highest electrical conduction and allowed investigating heat transport in 2D crystals [3]. Experimental measurements of the thermal conductivity of graphene were carried out in several research works [4,5,6,7,8,9]. The availability of high-quality few-layer graphene (FLG) led to experimental observations of the evolution of the thermal properties as the system dimensionality changed from 2D to 3D [6]. Several authors studied the thermal properties of graphene and showed its higher thermal conductivity [9,10,11,12,13].

Paz et al. [14] investigated the effect of an epitaxially grown graphene layer replacing the metallic contact over the active region in silicon carbide diodes as radiation detectors. Indeed, the surface properties of graphene usually play an important role in the practical application of graphene-based materials, especially in nanocomposites, nano-coating, and electrical nanodevices [15].

The effect of high temperatures on the structure of graphene and the changes in its morphology were studied by Amanda et al. [16]. The synthesis of graphene oxide (GO) through electrochemical oxidation of graphite and a reduction in electrochemically derived GO (EGO) were carried out by Xiong et al. [17], being easily transformed into highly crystalline graphene membranes. Graphene and graphene oxide (GO) were used to improve the properties of traditional cement-based building materials [18].

Kumuda et al. [19] explored several synthesis methods, structural distinctions, and a range of analytical techniques employed to compare properties among graphite, graphene oxide, and reduced graphene oxide (rGO). Some surface properties such as the surface energy of graphene and graphene oxide were studied by Wang et al. [20], obtaining results of 46.7 and 62.1 mJ/m^2^, respectively, while natural graphite flake presented a surface free energy of 54.8 mJ/m^2^ at room temperature.

The use of the IGC technique at infinite dilution to determine the surface properties of graphene oxide (GO) and graphene (rGO) was carried out for the first time by Dai et al. [21]. However, the results obtained by these authors were based on a hypothesis which considered the surface area and the London dispersive surface energy of the organic molecules as constant parameters. This failed in several research works [22,23,24,25,26], proving an important variation of the above parameters as a function of the temperature. Lee et al. [27] also determined the London dispersive and polar components as well as the Lewis acid-base properties of the surface free energy of graphene materials using the IGC technique at infinite dilution at room temperature. The same previous critiques can be formulated for the results obtained by Lee et al. [27], who limited their studies to only room temperature.

The lack of information and the different gaps encountered in this research area guided the objective of our study for accurate determination of the surface properties of graphene and carbon materials, which are required to obtain real guidance for the design and manufacturing of graphene-based biomaterials, medical instruments, structural composites, electronics, and renewable energy devices [20]. The present work is thus devoted to determination of the thermal surface properties, the London dispersive and polar acid-base surface energies, and the Lewis acid-base parameters of graphene and carbon materials as a function of the temperature by using the IGC technique [28,29,30,31,32,33,34,35,36,37,38,39,40,41,42,43,44,45,46,47,48,49,50,51,52,53,54,55,56,57,58,59,60,61,62,63,64,65,66,67,68,69,70,71,72,73] at infinite dilution and applying our new approach based on the Hamieh thermal model [22,23,24,25,26].

## 2. Results

### 2.1. London Dispersive Surface Energy of Solid Materials

The experimental results for the net retention volume $V_{n}$ of organic molecules adsorbed on the various solid surfaces as a function of the temperature T allowed giving the variations in $RTlnV_{n}(T)$ with the temperature for n-alkanes and polar molecules (Figure S1). An important variation was reported in the behavior of the $RTlnV_{n}$ of organic molecules adsorbed on the different solid substrates. The highest values were obtained in the case of graphene, followed by those of reduced graphene oxide. Meanwhile, the lowest values for $RTlnV_{n}$ were obtained with the untreated carbon fibers. Figure S1 also showed that the values obtained with graphene were 2.5 times greater than those of the untreated carbon fibers, thus proving a priori higher chemical activity for graphene relative to that of the carbon materials.

**Figure 1:**
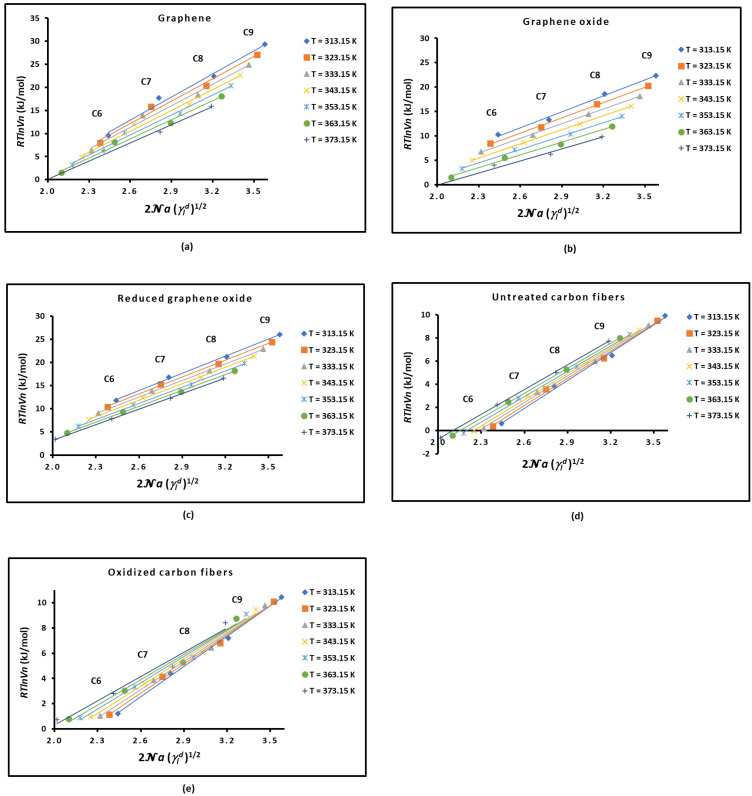
Variations in $RTlnV_{n}(T)$ as a function of $2Na(T){\left(\gamma_{l}^{d}\right)}^{1/2}$ of n-alkanes (from n-hexane (C6) to n-nonane (C9)) adsorbed on the different solid materials at different temperatures: (**a**) graphene, (**b**) graphene oxide, (**c**) reduced graphene oxide, (**d**) untreated carbon fibers, and (**e**) oxidized carbon fibers.

The obtained results for $RTlnV_{n}$ and the application of the Hamieh thermal model, which gave expressions of the surface area a(T) and the dispersive surface energy $\gamma_{l}^{d}(T)$ of organic molecules as a function of the temperature T [22,23,24,25,26], led to the representation of $RTlnV_{n}$ as a function of $2Na(T){\left(\gamma_{l}^{d}\right)}^{1/2}$ in Figure 1 for graphene and carbon materials.

**Figure 2:**
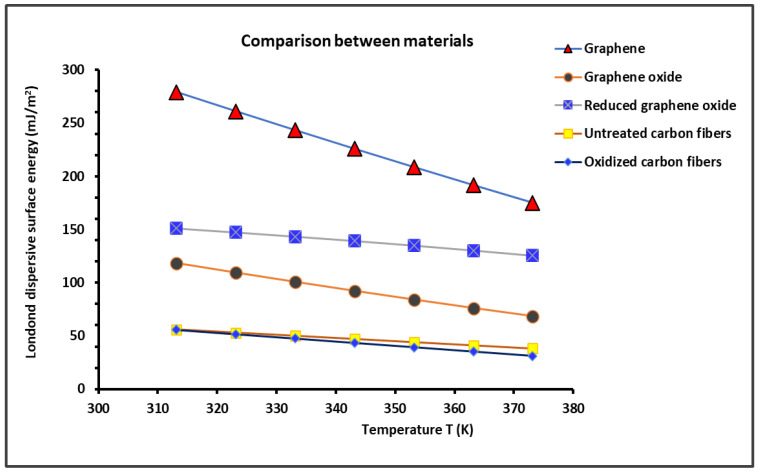
Evolution of the London dispersive surface energy of graphene and carbon materials as a function of the temperature.

The slope of the straight line of $RTlnV_{n}\left(T\right)=f\left[2Na(T)\;{\left(\gamma_{l}^{d}\right)}^{1/2}\right]$ obtained in Figure 1 for the different solid surfaces at various temperatures allowed an accurate determination of the London dispersive surface energy of $\gamma_{s}^{d}(T)$ for the various solids by using the Hamieh thermal model. The obtained results are plotted in Figure 2.

Figure 2 shows that the graphene exhibited the highest $\gamma_{s}^{d}(T)$ for all temperatures followed by the reduced graphene oxide, while the lowest $\gamma_{s}^{d}(T)$ was obtained in the case of the oxidized carbon fibers. The results showed a slight variation in $\gamma_{s}^{d}(T)$ between the untreated and oxidized carbon fibers. However, the oxidation of graphene considerably reduced the value of $\gamma_{s}^{d}(T)$ (about 50%) at 40 °C. This highlights the strong effect of the chemical structure on the value of the London dispersive surface energy of materials. Table 1 shows the differences between the various materials in the values of the London dispersive surface entropy $\varepsilon_{s}^{d}$, the extrapolated London dispersive surface energy at 0 K $\gamma_{s}^{d}(T=0\;K)$, and the temperature maximum $T_{Max}$.

A comparison between the results given in Table 1 and those in the literature [15,21,27,74,75,76,77] showed large differences in the values of the London dispersive surface energy. Dai et al. [21] in 2014 obtained for GO and rGO values of $\gamma_{s}^{d}$: 28.5 mJ/m^2^ and 98.3 mJ/m^2^ at 313.15 K, respectively. However, the same authors [74] in 2015 gave the following values: $\gamma_{s}^{d}$: 78.9 mJ/m^2^ and 106.8 mJ/m^2^ for the graphene oxide and reduced graphene oxide, respectively. Meanwhile, Lee et al. [27] obtained the results given in Table 2, which we compared to our results.

Table 2 shows that the deviation between the results of Dai et al. [21,74] and those obtained with the Hamieh thermal model varied from 33% to 76% for GO and from 29% to 35% for rGO, whereas the deviation with the results obtained by Lee et al. [27] was 7% for GO and 17% for rGO.

Lee et al. [27] gave the following variations in $\gamma_{s}^{d}(T)$ as a function of temperature in the interval of [300 K; 400 K]: $\gamma_{s}^{d}(T)$ = −0.540 T + 279.0 for graphene oxide and $\gamma_{s}^{d}\left(T\right)$ = −0.279T + 212.02 for reduced graphene oxide. These results also show an important variation in the values of the London dispersive surface entropy $\varepsilon_{s}^{d}$ and the extrapolated London dispersive surface energy $\gamma_{s}^{d}(T=0\;K)$ compared with those obtained using the Hamieh thermal model shown in Table 1. This large deviation between the results in the literature and those of the thermal model was certainly due to the fact that the effect of the temperature on the surface area of the organic molecules was neglected during calculation of the London dispersive surface energy of the materials.

**Figure 3:**
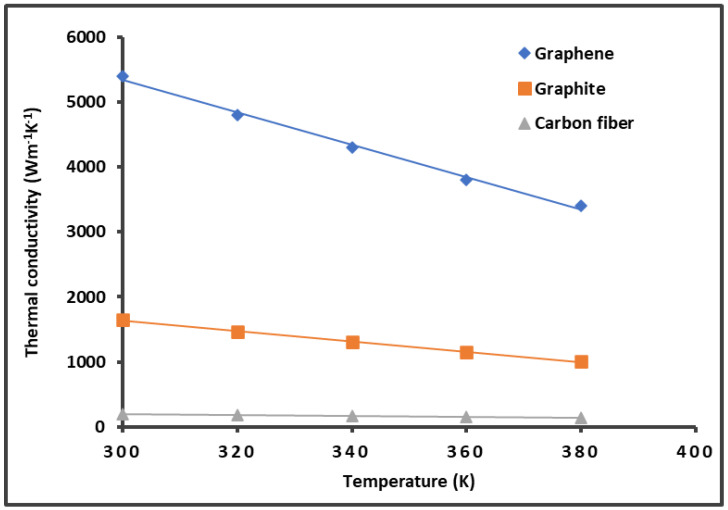
Variations in the thermal conductivity K of graphene and carbon materials as a function of the temperature.

On the other hand, the previous results shown in Figure 2 and Table 1 can be correlated to the thermal conductivity K of graphene and carbon materials. Figure 3 gives the variations in the thermal conductivity as a function of the temperature by using data in the literature [6,9,10,11,12,78,79,80,81,82,83,84]. In the studied temperature interval of [300 K; 400 K], it was observed that the thermal conductivity K decreased when the temperature increased for the graphene, graphite, and carbon fibers. The results in Figure 3 highlight the highest thermal conductivity of graphene, which also exhibited the highest London dispersive surface energy.

This result led us to see if there was a direct correlation between the thermal conductivity of material and its London dispersive surface energy not only for graphene and carbon materials but also for other metallic oxides, such as alumina, magnesium oxide, and zinc oxide. The values for the thermal conductivity were taken from the works of Hofmeister [82] and Wu et al. [83], whereas those for the London dispersive energy were obtained in previous studies [71,72].

**Figure 4:**
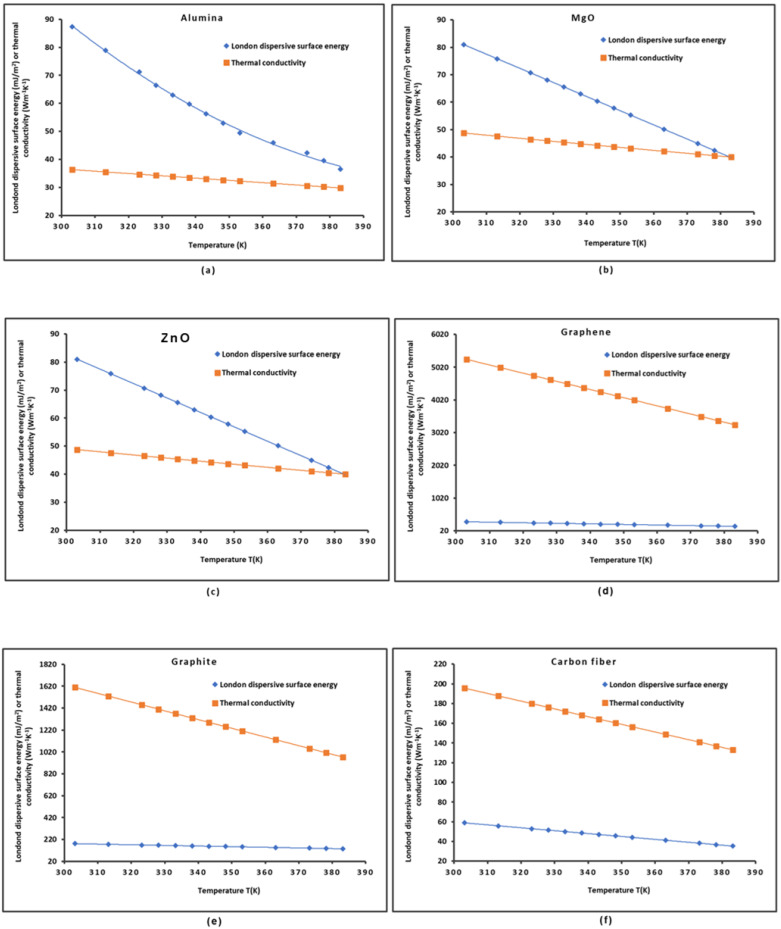
Evolution of the London dispersive surface energy $\gamma_{s}^{d}(T)$ and the thermal conductivity K(T) of alumina (**a**), MgO (**b**), ZnO (**c**), graphene (**d**), graphite (**e**), and carbon fibers (**f**) as a function of the temperature.

The variations in the London dispersive surface energy $\gamma_{s}^{d}(T)$ and the thermal conductivity K(T) of some solid materials such as alumina, MgO, ZnO, graphene, graphite, and carbon fibers as a function of the temperature are plotted in Figure 4.

The curves in Figure 4 all present a decrease in $\gamma_{s}^{d}(T)$ and K(T) when the temperature increased. The functions $\gamma_{s}^{d}(T)$ and K(T) were perfectly fitted with parabolic or linear curves with linear regression coefficients equal to 1.000. The highest values of $\gamma_{s}^{d}(T)$ and K(T) were obtained for graphene, showing the superiority of this material relative to the other materials for several applications.

**Figure 5:**
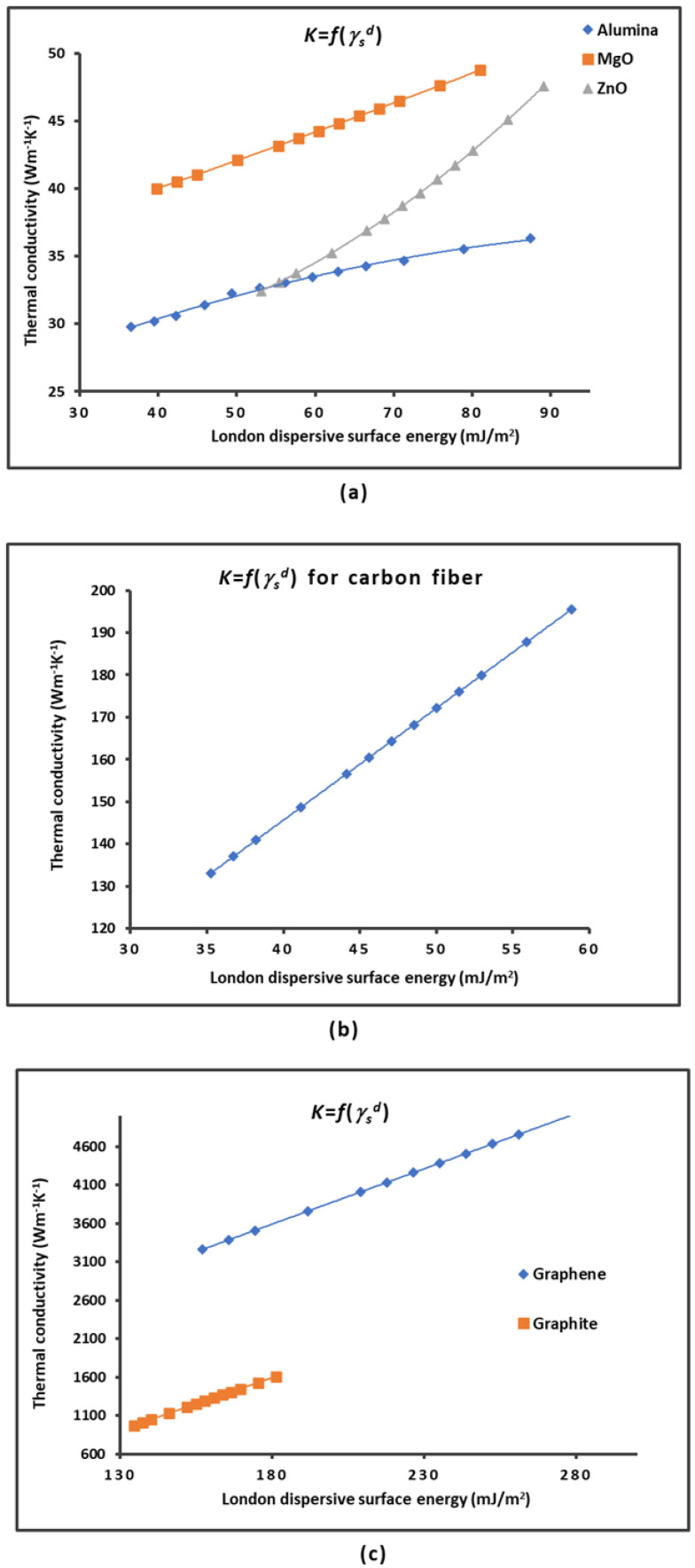
Variations in the thermal conductivity K(T) of alumina, MgO, and ZnO (**a**), carbon fibers (**b**), and graphene and graphite (**c**) as a function of the London dispersive surface energy $\gamma_{s}^{d}(T)$.

The variations in the thermal conductivity K(T) as a function of the London dispersive surface energy $\gamma_{s}^{d}(T)$ of different solid materials plotted in Figure 5 also showed parabolic or linear curves. Perfect linearity was assured for MgO, graphene, carbon fibers, and graphite, whereas perfect parabolic functions were obtained in the case of ZnO and alumina (Figure 5a) with linear regression coefficients equal to R^2^ = 1.000.

The results allowed us to give in Table 3 the various equations obtained for the different solid materials.

Table 3 and the curves in Figure 5 clearly show the important correlation between the thermal conductivity K(T) of the materials and the London dispersive surface energy $\gamma_{s}^{d}(T)$. When knowing one of these two parameters at a certain temperature, one can deduce the other. It can be concluded that the higher the London dispersive surface energy, the higher the thermal conductivity.

### 2.2. Lewis Acid-Base Properties of Solid Materials

Our new approach based on the London interaction energy was used to determine the free specific energy $-∆G_{a}^{p}\left(T\right)$ of interaction between the solid substrates and the different chosen polar organic solvents. The results are given in Table S1 for graphene, graphene oxide, reduced graphene oxide, untreated carbon fibers, and oxidized carbon fibers. It was observed that the graphene exhibited the highest polar free energy for all polar molecules, with greater values with basic solvents reflecting its highest acidic character.

**Figure 6:**
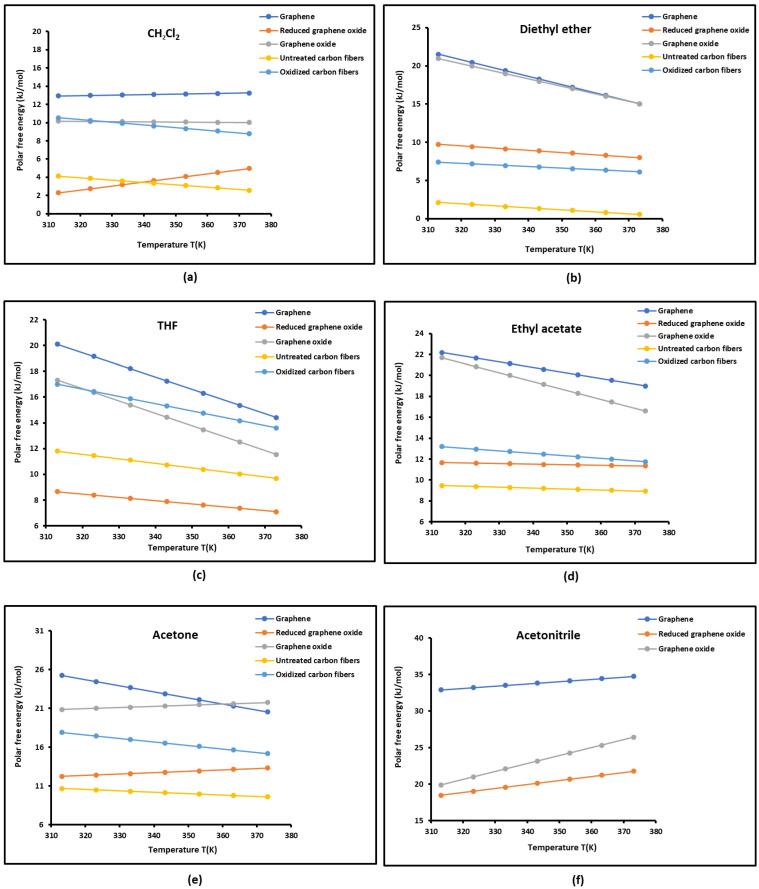
Variations in the polar free interaction energy $-∆G_{a}^{p}\left(T\right)$ of the various polar solvents adsorbed on the different graphene and carbon materials as a function of the temperature: (**a**) dichloromethane, (**b**) diethyl ether, (**c**) THF, (**d**) ethyl acetate, (**e**) acetone, and (**f**) acetonitrile.

The comparison between the different solid materials is elucidated in Figure 6. It can be deduced that the interaction energy was the highest with graphene followed by graphene oxide and reduced graphene oxide, which presented the lowest polar energy between the three graphenes. Figure 6 also shows that the reduced graphene oxide presented polar free energy values rather close to those of the untreated carbon fibers, whereas similar values were obtained for graphene oxide and oxidized carbon fibers in the case of the acid solvent.

This led to concluding that the oxidation of carbon materials gave similar interaction energy values, which was certainly due to the presence of identical surface groups such as carboxylate, hydroxyl, and epoxy. However, the gap between graphenes and carbon fibers increased in the case of basic or amphoteric polar molecules. This resulted in a difference in the acidic behavior of the different materials.

To quantify the acid-base behavior of different solid materials, it was necessary to determine the polar enthalpy ($-∆H_{a}^{p})$ and entropy ($-∆S_{a}^{p})$ values of the adsorbed polar molecules by using the variations in $-∆G_{a}^{p}\left(T\right)$ against the temperature. The results are given in Tables S2 and S3. They also show the highest polar enthalpy for graphene when interacting with the basic and amphoteric solvents, again demonstrating the highest Lewis acidity of graphene relative to the other graphenes and carbon materials.

The representation of $\left(\frac{-∆H_{a}^{p}}{{AN}^{\prime }}\right)$ and $\left(\frac{-∆S_{a}^{p}}{{AN}^{\prime }}\right)$ as a function of $\left(\frac{{DN}^{\prime }}{{AN}^{\prime }}\right)$ for the different polar solvents adsorbed on the solid surfaces led to the values of the Lewis enthalpic $K_{A}$ and $K_{D}$ and entropic $\omega_{A}$ and $\omega_{D}$ acid–base constants of the graphenes and carbon fibers. These acid-base values are given in Table 4. However, negative values were obtained for the Lewis acid-base constants on certain solid surfaces, such as graphene oxide, reduced graphene oxide, and oxidized carbon fibers. Meanwhile, the accepted values for graphene and the untreated carbon fibers showed the highest acidity for graphene occurring with a ratio *K_A_*/*K_D_* = 2.4 and highest basicity for the untreated carbon fibers with a ratio *K_A_*/*K_D_* = 0.7.

Nevertheless, the negative values of the acid-base constants indicates that the empirical relation $-∆H^{p}={DN\times K}_{A}+AN\times K_{D}$ used to calculate these constants should be corrected. In a previous paper [59], a new relation was proposed which takes into account the amphoteric effect by adding a new coupling acid-base constant $K_{CC}$ which corrects this gap: $-∆H^{p}=K_{A}\times DN+K_{D}\times AN-K_{CC}\times AN\times DN$. The resolution of such a linear system was developed in several previous works [26,58,72]. The results obtained by using this correction are included in Table 5.

Nevertheless, the negative values of the acid-base constants indicate that the empirical relation $-∆H^{p}={DN\times K}_{A}+AN\times K_{D}$ used to calculate these constants should be corrected. In a previous paper [58], a new relation was proposed which takes into account the amphoteric effect by adding a new coupling acid-base constant $K_{CC}$ which corrects this gap: $-∆H^{p}=K_{A}\times DN+K_{D}\times AN-K_{CC}\times AN\times DN$. The resolution of such a linear system was developed in several previous works [26,58,72]. The results obtained by using this correction are included in Table 5.

The corrected Lewis’s acid-base constants of the different solid materials led to positive values for $K_{A}$ and $K_{D}$ with a new coupling constant $K_{CC}$. The results in Table 5 allowed us to give the classification of the above materials in increasing order of their Lewis basicity as follows:

rGO ≤ GO < G < OCF < UCF

This implies that the untreated carbon fibers exhibited the highest basic character, whereas the lowest basicity was obtained with the reduced graphene oxide. Meanwhile, graphene had a slightly higher basic constant between the three graphenes, which admitted comparable basicity.

The comparison between the Lewis acidity strengths is given in increasing order of acid character as follows:

GO < UCF < OCF < G ≤ rGO

Indeed, the large difference between the graphene behaviors was due to the important variation in the acidic interaction force of these materials. Graphene and reduced graphene oxide exhibited the highest acidity, whereas graphene oxide showed the lowest acidic character. On the other hand, Table 5 highlights a higher K_D_/K_A_ ratio compared with other graphenes, which was certainly due to the presence of several surface basic groups such carboxylate, hydroxyl, and epoxy.

In conclusion, it was proven that graphene and reduced graphene oxide were more acidic than basic (in Lewis terms), whereas graphene and the carbon fibers exhibited more basic characteristics. These results are in perfect agreement with the surface nature of the various materials. The oxidation of graphene increased the basic characteristics of the material, while the reduction in graphene oxide increased the acid interaction force. The natural carbon fibers highlighted the highest basicity (about five times more basic than acidic).

The results obtained by Dai et al. [21] and Lee et al. [27] showed rather basic characteristics for reduced graphene oxide, contrary to the results of our present work. In fact, the values of the Lewis acid-base constants obtained by Dai et al. [21] and Lee et al. [27] were determined by neglecting the temperature’s effect on the surface area of organic molecules, which is necessary to use for an accurate determination of the polar enthalpy and then the acid-base constants of materials. On the other hand, the approach of Lee et al. [27] used the deformation polarizability of molecules without taking into account that of the solid substrates. The correction made by our new methodology led to accurate determination of the Lewis acid-base parameters of the different solid materials.

### 2.3. Polar Acid-Base Surface Energies of Graphenes and Carbon Fibers

The polar acid $\gamma_{s}^{+}$ and base $\gamma_{s}^{-}$ surface energies of the different solid materials were determined by using the method of Van Oss et al. [84], knowing the values of the polar free energy $-∆G_{a}^{sp}\left(T\right)$ of adsorbed solvents and their polar acid $\gamma_{l}^{+}$ and base $\gamma_{l}^{-}$ surface energies. The values of $-∆G_{a}^{sp}\left(T\right)$ are given in Table 6.

The values of the surface areas of the polar solvents obtained using the Hamieh thermal model and those of $-∆G_{a}^{sp}\left(T\right)$ in Table 6 led to the values of $\gamma_{s}^{+}$ and $\gamma_{s}^{-}$ for the different graphenes and carbon fibers and therefore the polar acid-base surface energy $\gamma_{s}^{p}=2\sqrt{\gamma_{s}^{+}\gamma_{s}^{-}}$. The total surface energy of the solid materials was obtained by summing the London dispersive and polar surface energy $\gamma_{s}^{tot.}={\gamma_{s}^{d}+\gamma }_{s}^{p}$. The results given in Table S4 show the highest values for the different components of the surface energy of graphene followed by those of oxidized carbon fibers, reduced graphene oxide, untreated carbon fibers, and graphene oxide. These results confirmed the highest values of $-∆G_{a}^{sp}\left(T\right)$ obtained for graphene surface.

**Figure 7:**
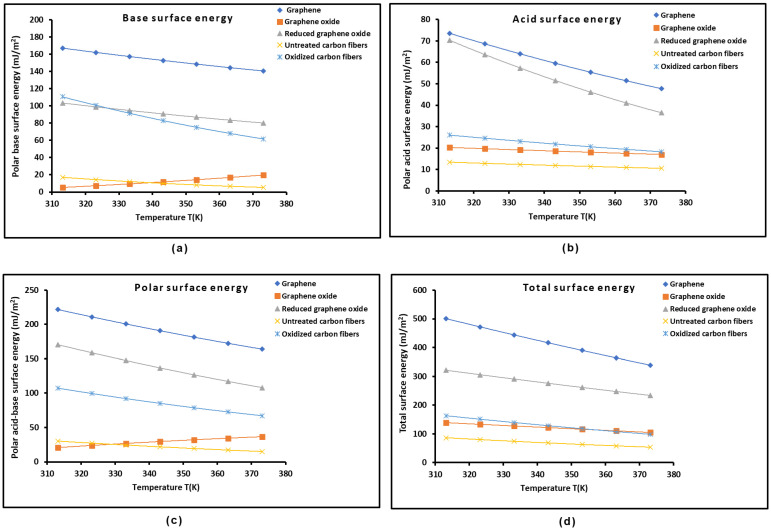
Evolution of polar acid-base energies $\gamma_{s}^{-}$ (**a**), $\gamma_{s}^{+}$ (**b**), and $\gamma_{s}^{p}$ (**c**) and total surface energy $\gamma_{s}^{tot.}$ (**d**) ($in\;mJ/m^{2}$) of the different graphenes and carbon materials against temperature.

The variations in the polar acid-base energies $\gamma_{s}^{+}$, $\gamma_{s}^{-}$, and $\gamma_{s}^{p}$ and the total surface energy $\gamma_{s}^{tot.}$ of the different graphenes and carbon materials as a function of the temperature are plotted in Figure 7. It can be easily observed that graphene exhibited the highest values for the different components of the surface energy, whereas reduced graphene oxide and oxidized carbon fibers exhibited close polar basic surface energy values, while the lowest basic surface energy was obtained with graphene oxide and untreated carbon fibers. The results of the acidic surface energy showed the highest value for graphene followed by reduced graphene oxide, whereas the lowest values were observed for the oxidized carbon fibers, graphene oxide, and untreated carbon fibers. The same conclusions were observed in Figure 7 for the polar and total surface energy of materials.

**Figure 8:**
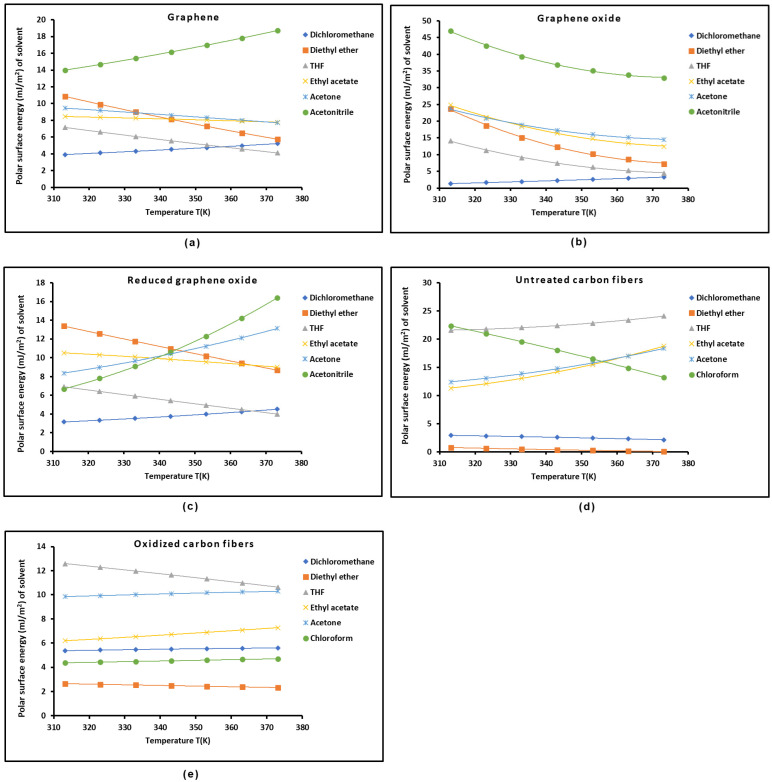
Variations in polar surface energy ($in\;mJ/m^{2}$) of different organic solvents adsorbed on graphene (**a**), graphene oxide (**b**), reduced graphene oxide (**c**), untreated carbon fibers (**d**), and oxidized carbon fibers (**e**) as a function of temperature.

The previous results led to the values for the polar surface energy of the different organic solvents using the Fowkes relation [85] and the values of the surface area of the molecules obtained from the Hamieh thermal model as a function of the temperature. The obtained results are given in curve form in Figure 8 for the different polar organic molecules adsorbed on the solid materials.

The polar surface energy of acetonitrile was shown in Figure 8 to be the highest for the three graphene surfaces. It was observed that the polar energy values of the different organic solvents were the highest in the case of graphene oxide and untreated carbon fibers. This result could not be considered separately. To conclude, it is necessary to determine the adhesion work of the polar molecules adsorbed on solid surfaces. The different results given in this work can help experts obtain interesting information on the adhesion work as a function of the temperature. Here, it can be mentioned that the new methodology proposed to quantify the surface properties of solid substrates using inverse gas chromatography will be quite useful for readers interested in determination of the acid-base properties and surface energy of materials.

### 2.4. Determination of the Average Separation Distance H

**Figure 9:**
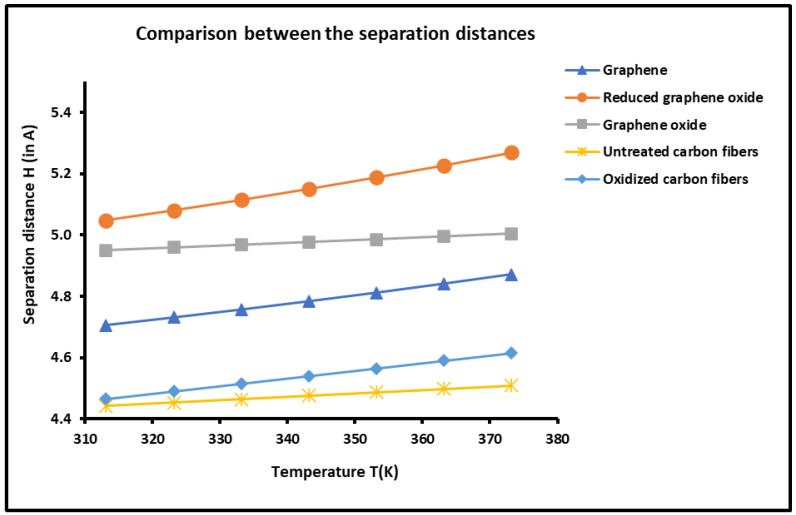
Variations in the average separation distance H(T) (in Å) between graphenes and carbon fibers, and organic molecules as a function of the temperature.

The experimental results shown in Figure S1 and Table S1 led to the determination of the average separation distance H between the solid graphenes and carbon fibers and organic molecules as a function of the temperature by using our new approach based on the London dispersion interaction. The results are given in Figure 9, which clearly show an effect of the temperature on the average separation distance. An increase in the distance H(T) versus the temperature was observed for all solid materials.

The thermal effect on the distance H(T), shown in Figure 9, led to classifying the graphenes and carbon fibers in decreasing order of H(T):

rGO > GO > G > OCF > UCF

This important result can be correlated with the interaction between materials and organic solvents. Indeed, when the attractive interaction force increased, the separation distance decreased. It seems that this result is in good agreement with those obtained with the Lewis acid-base properties. The above classification order of the distance H is the perfect inverse of the Lewis base constant $K_{D}$ for the graphenes and carbon fibers, which confirms that when the basicity of a solid surface decreases, the distance of the basic organic molecules increases.

## 3. Materials, Experiments, and Methods

### 3.1. Materials and Solvents

All chemicals such organic molecules, carbon fibers, graphene (G), graphene oxide (OG), and reduced graphene (rOG) solid materials used in this study were purchased from Fisher Scientific (Beirut, Lebanon). The non-polar solvents such as pentane, hexane, heptane, octane, nonane, and decane were utilized to determine the London dispersive properties of the different solid substrates. The polar solvents were used to determine the polar parameters of interaction with the solid surfaces. The Lewis acid molecules were as follows: carbon tetrachloride (CCl_4_), chloroform (CHCl_3_), and dichloromethane (CH_2_Cl_2_). The amphoteric solvents were acetone and acetonitrile, and the basic molecules were ethyl acetate, diethyl ether, and tetrahydrofuran (THF). The two carbon fibers, untreated fibers and oxidized fibers, were previously analyzed [71]. The corrected acceptor number and normalized donor number of the electrons of the polar solvents were given in other papers [74,75,76].

### 3.2. Experiments

Experimental measurements were performed on a commercial Focus GC gas chromatograph (See Figure S2) equipped with a flame ionization detector (Sigma-Aldrich, St. Quentin Fallavier, France). The solid particles were poured into a stainless steel column with a 2 mm inner diameter and a length of 20 cm. The temperature range varied from 40 °C to 100 °C. The column was packed with 1 g of solid particles. The standard deviation of the obtained retention time *t_R_* was less than 1% in all measurements. The temperatures of the injector and detector were fixed at 200 °C. Infinite dilution of the probes was satisfied by using 1 µL Hamilton syringes and injecting extremely small quantities of the vapor probe, satisfying the limit of detection of the FID of high sensitivity (Figure S2) to practically realize the zero-surface coverage [59,72]. The columns containing the solid particles were preconditioned at 130 °C overnight to ensure the total desorption of water molecules or any other residual impurities [59,72]. This technique is called inverse gas chromatography (IGC) at infinite dilution. The n-alkanes and polar solvents were injected into the column containing the solid particles or fibers. The retention times of these probes, measured at infinite dilution, allowed us to determine the interactions between the organic molecules and the solids by supposing that there was no lateral interaction between the probe molecules themselves. Experimental measurements led to the values of the retention time $t_{R}$ of the injected organic solvents and the retention time $t_{0}$ of a non-adsorbing probe such as methane, which is called the dead reference retention time. The net retention volume *V_n_* of the probes was consequently calculated from the following relation:

(1) $$V_{n}=jD\left(t_{R}-t_{0}\right)$$

where *D* is the flow rate of the carrier gas (helium) and *j* is a correction factor which takes into account the compression of the gas.

The experimental values of the net retention volume $V_{n}$ led to variations in $RTlnV_{n}$ for the different organic molecules adsorbed on the various solid materials as a function of the temperature. The obtained results were given in Table 7.

### 3.3. Methods and Models

The chromatographic experiments giving the values of $RTlnV_{n}(T)$ for the adsorbed organic molecules led to variations in the free energy $∆G_{a}^{0}$ for the adsorption of solvents on the various solid materials using the following equation:

(2) $$∆G_{a}^{0}=-RTlnV_{n}+B\left(T\right),$$

where *T* is the absolute temperature, *R* the perfect gas constant, and B(T) is a constant depending on the temperature and the two-dimensional reference state of the adsorbed film.

The surface variable $∆G_{a}^{0}$ can be written as follows:

(3) $$∆G_{a}^{0}=∆G_{a}^{d}+∆G_{a}^{p},$$

where $∆G_{a}^{d}$ and $∆G_{a}^{p}$ are the London dispersion component and the polar component of the free energy adsorption, respectively.

Many methods or models were used in the literature [40,65,66,67,69,70] to separate the London dispersive and polar contributions of the total free energy of interaction. However, it was proven in several recent studies that these methods cannot be used quantitatively due to some gaps, incoherencies, and irregularities in their concepts and applications. We proposed a more rigorous method for separation of the London dispersive and polar terms based on the London dispersive interaction energy between the solvents and the solid surfaces [72,73].

The determination of the London dispersive component $\gamma_{s}^{d}$ of the surface energy of solid materials was also corrected by using the Hamieh thermal model [22,23,24,25,26] and taking into account the temperature effect on the surface area of organic molecules and the London dispersive component $\gamma_{l}^{d}$ of the surface energy of solvents.

The London dispersion free energy can be expressed as follows:

(4) $$∆G_{a}^{d}\left(T\right)=-\frac{\alpha_{0S}\;}{\;H^{6}}\left[\frac{3N}{{2\left(4\pi \varepsilon_{0}\right)}^{2}}\left(\frac{\varepsilon_{S}\;\varepsilon_{X}}{\left(\varepsilon_{S}+\varepsilon_{X}\right)}\alpha_{0X}\right)\right],$$

where N is Avogadro’s number, $\varepsilon_{0}$ is the permittivity of a vacuum, *S* denotes the solid particle, *X* is the solvent molecule separated by a distance H, and $\varepsilon_{S}$ and $\varepsilon_{X}$ are the respective ionization energies of the solid S and the solvent X, respectively. The new parameter of interaction taken for the quantification of the polar free energy of interaction is given by

(5) $$P_{SX}=\frac{\varepsilon_{S}\;\varepsilon_{X}}{\left(\varepsilon_{S}+\varepsilon_{X}\right)}\alpha_{0X}$$

For the n-alkanes $\left(C_{n}\right)$ adsorbed on the solid material, the energetic parameter $RTlnVn\left(C_{n}\right)$ of adsorption is expressed by

(6) $$RTlnVn\left(C_{n}\right)=A\left[\frac{3N}{{2\left(4\pi \varepsilon_{0}\right)}^{2}}P_{SX}\left(C_{n}\right)\right]-B.$$

The constant A is given by

(7) $$A=\frac{\alpha_{0S}\;}{\;H^{6}}.$$

The representation of $RTlnVn\left(C_{n}\right)$ against $\frac{3N}{{2\left(4\pi \varepsilon_{0}\right)}^{2}}P_{SX}\left(C_{n}\right)$ gave a straight line of n-alkanes, allowing the determination of the polar free energy $\left(-∆G_{a}^{p}(T)\right)$ of adsorbed polar solvents as a function of the temperature using the following equation:

(8) $$-∆G_{a}^{p}\left(T\right)=RTlnVn\left(X\right)-A\left[\frac{3N}{{2\left(4\pi \varepsilon_{0}\right)}^{2}}P_{S-X}\right]+B$$

This led to the polar enthalpy $-∆H_{a}^{p}(T)$ and entropy $-∆S_{a}^{p}(T)$ of organic molecules using the following thermodynamic relations:

(9) $$\left\{\begin{array}{c} ∆H_{a}^{p}\left(T\right)=\frac{\partial \left(\frac{∆G_{a}^{p}\left(T\right)}{T}\right)}{\partial \left(\frac{1}{T}\right)}\;\\ ∆S_{a}^{p}\left(T\right)=-\left(\frac{\partial \left(∆G_{a}^{p}\left(T\right)\right)}{\partial T}\right) \end{array}\right.$$

The values of $-∆H_{a}^{p}(T)$ and $-∆S_{a}^{p}(T)$ for the adsorbed polar solvents were obtained as a function of the temperature, and this allowed obtaining the Lewis enthalpic acid-base constants $K_{A}$ and $K_{D}$ and the entropic acid-base parameters $\omega_{A}$ and $\omega_{D}$:

(10) $$\left\{\begin{array}{c} -∆H^{p}={DN\times K}_{A}+AN\times K_{D}\\ -∆S^{p}={DN\times \omega }_{A}+AN\times \omega_{D} \end{array}\right.$$

where *AN* and *DN* are the Gutmann electron donor and acceptor numbers of the polar solvents, respectively [86]. The used values were those corrected by Riddle and Fowkes [87].

The surface energy of the solid surfaces can be written as

(11) $$\gamma_{s}=\gamma_{s}^{d}+\gamma_{s}^{p}$$

where $\gamma_{s}^{p}$ represents the total polar (or acid-base) contribution $\gamma_{s}^{p}$ of the surface energy.

The determination of $\gamma_{s}^{d}(T)$ for the various solid materials was determined using the Fowkes relation [85] and the Hamieh thermal model, giving the surface area a(T) of organic molecules as a function of the temperature [22,23,24,25,26]:

(12) $$RTlnV_{n}=2Na(T){\left(\gamma_{l}^{d}\gamma_{s}^{d}\right)}^{1/2}+\alpha (T)$$

where α(T) is a constant depending only on the temperature and the solid material.

To determine $\gamma_{s}^{p}$ for the different solid surfaces, we applied Van Oss et al.’s method [84], which consists of the determination of the Lewis acid $\gamma_{s}^{+}$ and base $\gamma_{s}^{-}$ surface energies of the solids first. Upon knowing the Lewis acid $\gamma_{l}^{+}$ and base $\gamma_{l}^{-}$ surface energies of the used solvents, the value of $\gamma_{s}^{p}$ will therefore be determined. To accomplish this, two monopolar solvents, ethyl acetate (*B*) and dichloromethane (*A*), were used by Van Oss et al. [84]. They are characterized as follows:

(13) $$\left\{\begin{array}{c} \gamma_{A}^{+}=5.2\frac{mJ}{m^{2}},\;\gamma_{A}^{-}=0\\ \gamma_{B}^{+}=0,\;\gamma_{B}^{-}=19.2\;mJ/m^{2} \end{array}\right.$$

Knowing the polar free energy $∆G_{a}^{sp}\left(T\right)$ of the polar molecules, Van Oss et al. proposed the following relation [87]:

(14) $$∆G_{a}^{p}\left(T\right)=2Na(T)\left(\sqrt{\gamma_{l}^{-}\gamma_{s}^{+}}+\sqrt{\gamma_{l}^{+}\gamma_{s}^{-}}\right)$$

The Lewis acid and base surface energies of the solid surfaces are then given by

(15) $$\left\{\begin{array}{c} \begin{array}{c} \gamma_{s}^{+}\left(T\right)=\frac{{\left[∆G_{a}^{p}\left(T\right)\left(B\right)\right]}^{2}}{{4N}^{2}{\left[a_{B}(T)\right]}^{2}\gamma_{B}^{-}}\; \end{array}\;\\ \gamma_{s}^{-}\left(T\right)=\frac{{\left[∆G_{a}^{p}\left(T\right)\left(A\right)\right]}^{2}}{{4N}^{2}{\left[a_{A}(T)\right]}^{2}\gamma_{A}^{+}}\; \end{array}\right.$$

This led to the polar (or acid-base) surface energy ${\gamma_{s}^{p\;}=\gamma }_{s}^{AB}$ and the total surface energy $\gamma_{s}^{tot.}$ of different materials:

(16) $$\left\{\begin{array}{c} \gamma_{s}^{AB}=2\sqrt{\gamma_{s}^{+}\gamma_{s}^{-}}\\ \gamma_{s}^{tot.}={\gamma_{s}^{d}+\gamma }_{s}^{AB} \end{array}\right.$$

## 4. Conclusions

The application of our new approach, consisting of the use of the London dispersion interaction, the Hamieh thermal model, and the model of coupling the amphoteric constant, under inverse gas chromatography at infinite dilution led to accurate determination of the surface properties of graphene, graphene oxide, reduced graphene oxide, untreated carbon fibers, and oxidized carbon fibers. Indeed, the London dispersion equation allowed separation of the dispersive and polar contributions of the free energy of adsorption of organic molecules on graphenes and carbon materials, and consequently, this led to quantification of the polar surface thermodynamic variables and acid-base constants of these materials. Meanwhile, the Hamieh thermal model conducted a better determination of the London dispersive surface energy, the polar surface energy, the acid and base surface energies of solid surfaces, as well as the polar surface energy of the organic solvents adsorbed on graphenes and carbon fibers. Furthermore, the model of the third amphoteric constant corrected the values of the Lewis acid-base constants of solid materials. A successful first attempt consisted of correlating the thermal conductivity of graphenes and carbon fibers with their London dispersive surface energy. It was proven that graphene exhibited the highest thermal conductivity and also the highest London dispersive and polar surface energies. This new approach corrected some serious errors and deficiencies made in the literature by some authors. This study gives, for the first time, the variations in polar surface energy of graphenes as well as the acid and base surface energies as a function of the temperature. The various results obtained in this work all converged toward the final conclusion of the highest polar and dispersive characteristics of graphene and it highest Lewis acid-base behavior.

## Figures and Tables

**Table 1:** The $\gamma_{s}^{d}(T)$ equations of graphene, graphene oxide, reduced graphene oxide, and carbon fibers (untreated and oxidized), with the London dispersive surface entropy $\varepsilon_{s}^{d}$, the extrapolated London dispersive surface energy at 0 K $\gamma_{s}^{d}(T=0\;K)$, and the temperature maximum $T_{Max}$.

Solid Material	$\gamma_{s}^{d}(T)$ (mJ/m^2^)	$\varepsilon_{s}^{d}={d\gamma }_{s}^{d}/dT$ (mJ m^−2^ K^−1^)	$\gamma_{s}^{d}(T=0\;K)$ (mJ/m^2^)	$T_{Max}$ (K)
Graphene	$\gamma_{s}^{d}(T)$ = −1.736T + 822.22	−1.736	822.22	473.5
Graphene oxide	$\gamma_{s}^{d}(T)$ = −0.832T + 377.98	−0.832	377.98	454.6
Reduced graphene oxide	$\gamma_{s}^{d}\left(T\right)\;$= −0.424T + 284.29	−0.424	284.29	670.5
Untreated carbon fibers	$\gamma_{s}^{d}(T)$ = −0.295T + 148.22	−0.295	148.22	502.8
Oxidized carbon fibers	$\gamma_{s}^{d}(T)$ = −0.409T + 183.60	−0.409	183.60	449.4

**Table 2:** Comparison between the values obtained in this work compared to those in the literature for the London dispersive surface energy $\gamma_{s}^{d}$ of graphene oxide and reduced graphene oxide at 313.15 K.

Solid Material	$\gamma_{s}^{d}(T)$ (mJ/m^2^) Lee et al. [27]	$\gamma_{s}^{d}(313.15\;K)$ (mJ/m^2^) Dai et al. [21]	$\gamma_{s}^{d}(313.15\;K)$ (mJ/m^2^) Dai et al. [74]	$\gamma_{s}^{d}(313.15\;K)$ (mJ/m^2^) This Work
Graphene oxide	110	28.5	78.9	118.2
Reduced graphene oxide	125	98.3	106.8	151.0

**Table 3:** Equations for the thermal conductivity of materials as a function of the London dispersive surface energy $K=f\left(\gamma_{s}^{d}\right)$.

Material	$\mathrm{Equation}\;K=f\left(\gamma_{s}^{d}\right)$
MgO	K = 0.2124 $\gamma_{s}^{d}$ + 31.47
Graphene	K = 14.398 $\gamma_{s}^{d}$ + 1002
Graphite	K = 13.613 $\gamma_{s}^{d}$ + 856.09
Carbon fibers	K = 2.646 $\gamma_{s}^{d}$ + 39.83
Alumina	K = −0.0012 $\left(\gamma_{s}^{d}\right)$^2^ + 0.281 $\gamma_{s}^{d}$ + 21.123
ZnO	K = 0.004 $\left(\gamma_{s}^{d}\right)$^2^ − 0.142 $\gamma_{s}^{d}$ + 28.794

**Table 4:** Values of the acid-base constants $K_{A}$, $K_{D}$, $\omega_{A}$, and $\omega_{D}\;$ and linear regression coefficient *R*^2^ of graphenes and carbon fibers with the corresponding acid-base ratios.

Material	*K_D_*	*K_A_*	*K_D_*/*K_A_*	R^2^	10^−3^*ω_A_*	10^−3^*ω_D_*	*ω_D_*/*ω_A_*	R^2^
Graphene	0.253	0.593	0.426	0.9906	−1.346	1.187	−1.134	0.9563
Graphene oxide	−0.551	0.223	−2.471	0.9833	−3.305	0.416	−7.951	0.9412
Reduced graphene oxide	−0.721	0.601	−1.200	0.9608	−1.800	1.232	−1.461	0.9421
Untreated carbon fibers	0.345	0.235	1.468	0.8465	0.927	0.348	2.663	0.7631
Oxidized carbon fibers	−0.010	0.381	−0.025	0.9120	−0.613	0.630	−0.973	0.9002

**Table 5:** Corrected values of the acid-base constants $K_{A}$, $K_{D}$, and new amphoteric constant *K* of graphenes and carbon fibers with the corresponding acid-base ratios *K_D_*/*K_A_*.

Material	*K_D_*	*K_A_*	$K_{CC}$	*K_D_*/*K_A_*
Graphene (G)	0.278	0.594	5.7 × 10^−4^	0.468
Graphene oxide (GO)	0.227	0.069	−7.9 × 10^−3^	3.306
Reduced graphene oxide (rGO)	0.217	0.631	2.1 × 10^−2^	0.344
Untreated carbon fibers (UCFs)	1.587	0.325	4.4 × 10^−2^	4.883
Oxidized carbon fibers (OCFs)	1.190	0.468	4.2 × 10^−2^	2.543

**Table 6:** Values of $-∆G_{a}^{sp}\left(T\right)\;(in\;kJ/mol)$ for dichloromethane and ethyl acetate adsorbed on solid materials at different temperatures.

Dichloromethane					
T (K)	G	GO	rGO	UCF	OCF
313.15	12.938	2.309	10.175	4.130	10.526
323.15	12.993	2.749	10.148	3.872	10.235
333.15	13.048	3.192	10.123	3.614	9.944
343.15	13.102	3.639	10.095	3.356	9.653
353.15	13.157	4.082	10.068	3.098	9.362
363.15	13.211	4.523	10.042	2.840	9.071
373.15	13.265	4.964	10.013	2.582	8.780
**Ethyl Acetate**					
**T (K)**	**G**	**GO**	**rGO**	**UCF**	**OCF**
313.15	22.191	11.657	21.696	9.471	13.204
323.15	21.658	11.603	20.849	9.381	12.962
333.15	21.125	11.552	20.000	9.291	12.720
343.15	20.591	11.504	19.151	9.201	12.478
353.15	20.057	11.452	18.302	9.111	12.236
363.15	19.524	11.400	17.453	9.021	11.994
373.15	18.990	11.347	16.604	8.931	11.752

**Table 7:** Values of $RTlnV_{n}\left(T\right)$ (kJ/mol) for organic molecules adsorbed on the various solid materials against the temperature. Graphene, graphene oxide, reduced graphene oxide, untreated carbon fibers, and oxidized carbon fibers.

$RTlnV_{n}\left(T\right)$ (kJ/mol)		Graphene					
Solvents	313.15 K	323.15 K	333.15 K	343.15 K	353.15 K	363.15 K	373.15 K
n-hexane	9.560	7.950	6.340	4.730	3.120	1.510	−0.100
n-heptane	17.765	15.850	13.935	12.020	10.105	8.190	6.275
n-octane	22.360	20.350	18.340	16.330	14.320	12.310	10.300
n-nonane	29.361	27.116	24.871	22.626	20.381	18.136	15.891
CH_2_Cl_2_	6.948	5.905	4.862	3.819	2.776	1.733	0.690
Diethyl ether	13.313	12.025	10.737	9.449	8.161	6.873	5.585
THF	17.305	15.025	12.745	10.465	8.185	5.905	3.625
Ethyl acetate	20.285	18.000	15.715	13.430	11.145	8.860	6.575
Acetone	11.920	10.375	8.830	7.285	5.740	4.195	2.650
Acetonitrile	10.070	8.425	6.780	5.135	3.490	1.845	0.200
$RTlnV_{n}\left(T\right)$ **(kJ/mol)**		**Graphene oxide**					
Solvents	313.15 K	323.15 K	333.15 K	343.15 K	353.15 K	363.15 K	373.15 K
n-hexane	10.250	8.500	6.750	5.000	3.250	1.500	−0.250
n-heptane	13.284	11.734	10.184	8.634	7.084	5.534	3.984
n-octane	18.550	16.500	14.450	12.400	10.350	8.300	6.250
n-nonane	22.329	20.245	18.162	16.079	13.996	11.913	9.829
CH_2_Cl_2_	3.750	2.855	1.960	1.065	0.170	−0.725	−1.620
Diethyl ether	5.682	4.748	3.814	2.880	1.946	1.012	0.078
THF	10.085	8.534	6.983	5.432	3.881	2.330	0.779
Ethyl acetate	11.914	10.281	8.648	7.015	5.382	3.749	2.116
Acetone	8.040	6.876	5.712	4.548	3.384	2.220	1.056
Acetonitrile	5.400	4.587	3.774	2.961	2.148	1.335	0.522
$RTlnV_{n}\left(T\right)$ **(kJ/mol)**		**Reduced graphene oxide**					
Solvents	313.15 K	323.15 K	333.15 K	343.15 K	353.15 K	363.15 K	373.15 K
n-hexane	11.800	10.400	9.000	7.600	6.200	4.800	3.400
n-heptane	16.800	15.300	13.800	12.300	10.800	9.300	7.800
n-octane	21.200	19.700	18.200	16.700	15.200	13.700	12.200
n-nonane	25.998	24.433	22.865	21.299	19.733	18.165	16.599
CH_2_Cl_2_	8.491	7.170	5.849	4.528	3.207	1.886	0.565
Diethyl ether	14.820	13.450	12.080	10.710	9.340	7.970	6.600
THF	22.199	19.905	17.611	15.317	13.023	10.729	8.435
Ethyl acetate	21.830	19.525	17.220	14.915	12.610	10.305	8.000
Acetone	18.485	16.375	14.265	12.155	10.045	7.935	5.825
Acetonitrile	13.999	12.930	11.861	10.792	9.723	8.654	7.585
$RTlnV_{n}\left(T\right)$ **(kJ/mol)**		**Untreated carbon fibers**					
Solvents	313.15 K	323.15 K	333.15 K	343.15 K	353.15 K	363.15 K	373.15 K
n-hexane	0.591	0.388	0.185	-0.018	−0.221	−0.424	−0.627
n-heptane	3.859	3.589	3.319	3.049	2.779	2.509	2.239
n-octane	6.487	6.244	6.001	5.758	5.515	5.272	5.029
n-nonane	9.917	9.494	9.101	8.686	8.285	7.984	7.734
CCl_4_	1.830	1.760	1.690	1.626	1.564	1.500	1.442
CH_2_Cl_2_	−2.639	−2.911	−3.212	−3.517	−3.851	−4.279	−4.787
CHCl_3_	11.181	10.463	9.653	8.741	7.642	6.137	3.682
Diethyl ether	−1.970	−2.305	−2.668	−3.019	−3.389	−3.848	−4.362
THF	5.562	5.107	4.651	4.197	3.743	3.282	2.820
Benzene	5.709	5.441	5.183	4.919	4.659	4.428	4.213
Ethyl acetate	5.326	5.092	4.860	4.634	4.412	4.191	3.982
Acetone	1.539	1.308	1.065	0.841	0.616	0.348	0.071
$RTlnV_{n}\left(T\right)$ **(kJ/mol)**		**Oxidized carbon fibers**					
Solvents	313.15 K	323.15 K	333.15 K	343.15 K	353.15 K	363.15 K	373.15 K
n-hexane	1.211	1.143	1.061	0.979	0.897	0.815	0.733
n-heptane	4.409	4.139	3.869	3.599	3.329	3.059	2.789
n-octane	7.192	6.814	6.436	6.058	5.680	5.302	4.924
n-nonane	10.445	10.107	9.769	9.431	9.093	8.755	8.417
CCl_4_	3.505	3.427	3.341	3.256	3.173	3.090	3.009
CH_2_Cl_2_	4.441	4.238	4.035	3.832	3.629	3.426	3.223
CHCl_3_	9.763	9.484	9.205	8.926	8.647	8.368	8.089
Diethyl ether	3.941	3.738	3.535	3.332	3.129	2.926	2.723
THF	11.359	10.873	10.379	9.886	9.394	8.902	8.411
Benzene	8.182	7.919	7.652	7.386	7.120	6.855	6.590
Ethyl acetate	9.662	9.432	9.202	8.972	8.742	8.512	8.282
Acetone	9.379	9.082	8.785	8.488	8.191	7.894	7.597

## Data Availability

The data presented in this study are available in the article and Supplementary Materials.

## Supplementary Materials

The following supporting information can be downloaded at: https://www.mdpi.com/article/10.3390/molecules29122871/s1, Figure S1: Variations in $RTlnV_{n}(T)$ for n-alkanes and polar molecules adsorbed on the various solid materials against the temperature. (a) Graphene, (b) graphene oxide, (c) reduced graphene oxide, (d) untreated carbon fibers, and (e) oxidized carbon fibers. Table S1: Values of $-∆G_{a}^{p}\left(T\right)$ (in kJ/mol) for polar molecules adsorbed on the various solid materials against the temperature, showing graphene, graphene oxide, reduced graphene oxide, untreated carbon fibers, and oxidized carbon fibers. Table S2: Values of ($-∆H_{a}^{p}\;in\;kJ\;{mol}^{-1}$) for polar molecules adsorbed on the different graphene and carbon materials. Table S3: Values of ($-∆S_{a}^{p}\;in\;J\;{K^{-1}mol}^{-1}$) for polar molecules adsorbed on the different graphene and carbon materials. Table S4: Values of polar acid-base energies $\gamma_{s}^{+}$, $\gamma_{s}^{-}$, and $\gamma_{s}^{p}$ and total surface energy $\gamma_{s}^{tot.}$ ($in\;mJ/m^{2}$) of the different graphenes and carbon materials at various temperatures. Figure S2: Schematic diagram of a gas chromatograph.
