# Supplementary material for: Thermal Surface Properties, London Dispersive and Polar Surface Energy of Graphene and Carbon Materials Using Inverse Gas Chromatography at Infinite Dilution
Source: Molecules. 2024 Jun 17;29(12):2871. doi: 10.3390/molecules29122871 (PMC11206878; doi:10.3390/molecules29122871)
Supplement: Supplementary file 1 [file molecules-29-02871-s001.zip › molecules-3048817-supplementary.pdf]

Support information

Surface Thermal Properties, London Dispersive and Polar Surface Energy of Graphene and Carbon Materials using Inverse Gas Chromatography at Infinite Dilution

Tayssir Hamieh[1,2]

[1]Faculty of Science and Engineering, Maastricht University, P.O. Box 616, 6200 MD Maastricht, The Netherlands; t.hamieh@maastrichtuniversity.nl

[2]Laboratory of Materials, Catalysis, Environment and Analytical Methods (MCEMA), Faculty of Sciences, Lebanese University, Beirut P.O. Box 6573/14, Lebanon

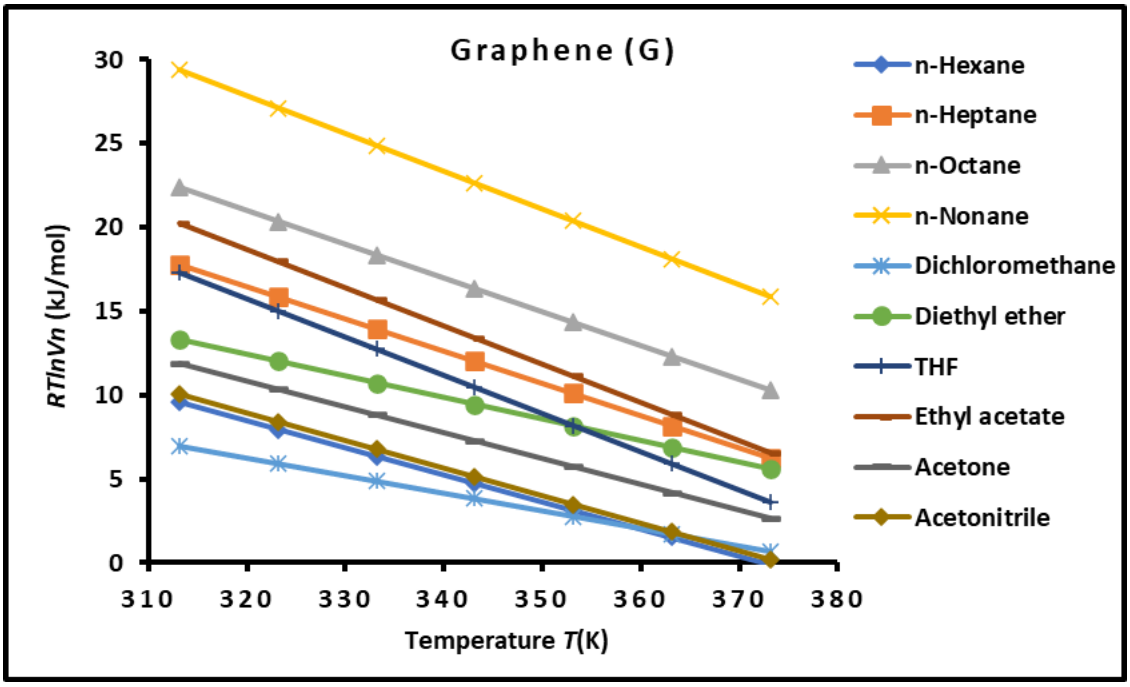

(a)

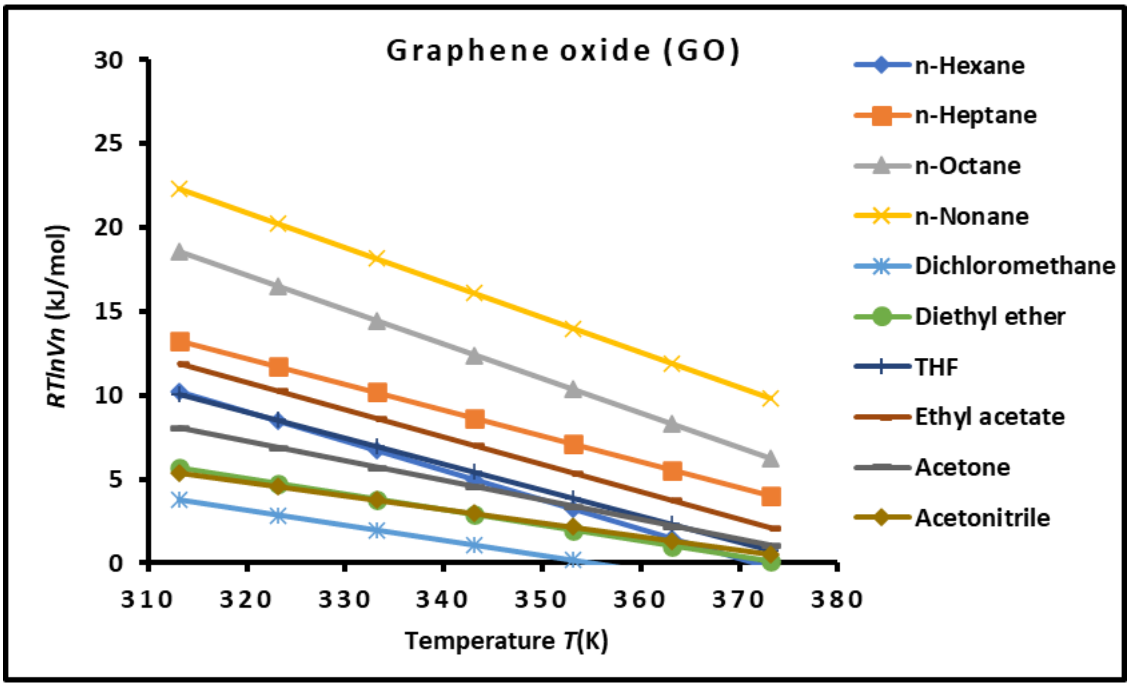
Graphene oxide (GO)
RTlnVn (kJ/mol)
Temperature T(K)
0
5
10
15
20
25
30
310
320
330
340
350
360
370
380
n-Hexane
n-Heptane
n-Octane
n-Nonane
Dichloromethane
Diethyl ether
THF
Ethyl acetate
Acetone
Acetonitrile

(b)

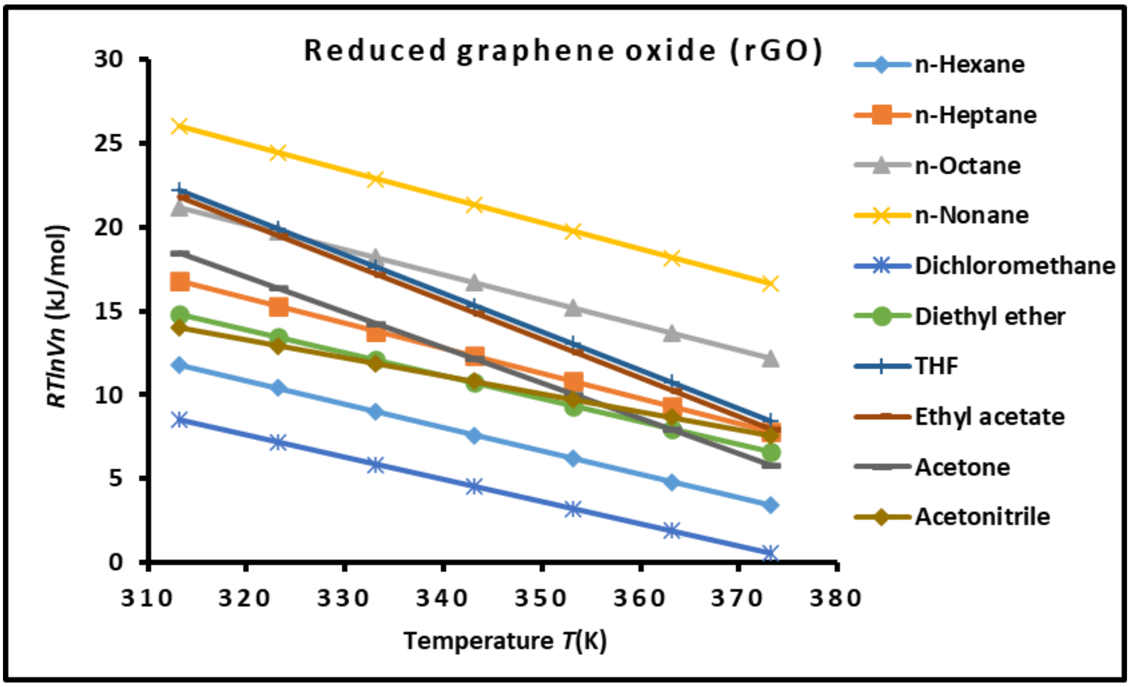
Reduced graphene oxide (rGO)
RTlnVn (kJ/mol)
Temperature T(K)
0
5
10
15
20
25
30
310
320
330
340
350
360
370
380
n-Hexane
n-Heptane
n-Octane
n-Nonane
Dichloromethane
Diethyl ether
THF
Ethyl acetate
Acetone
Acetonitrile

(c)

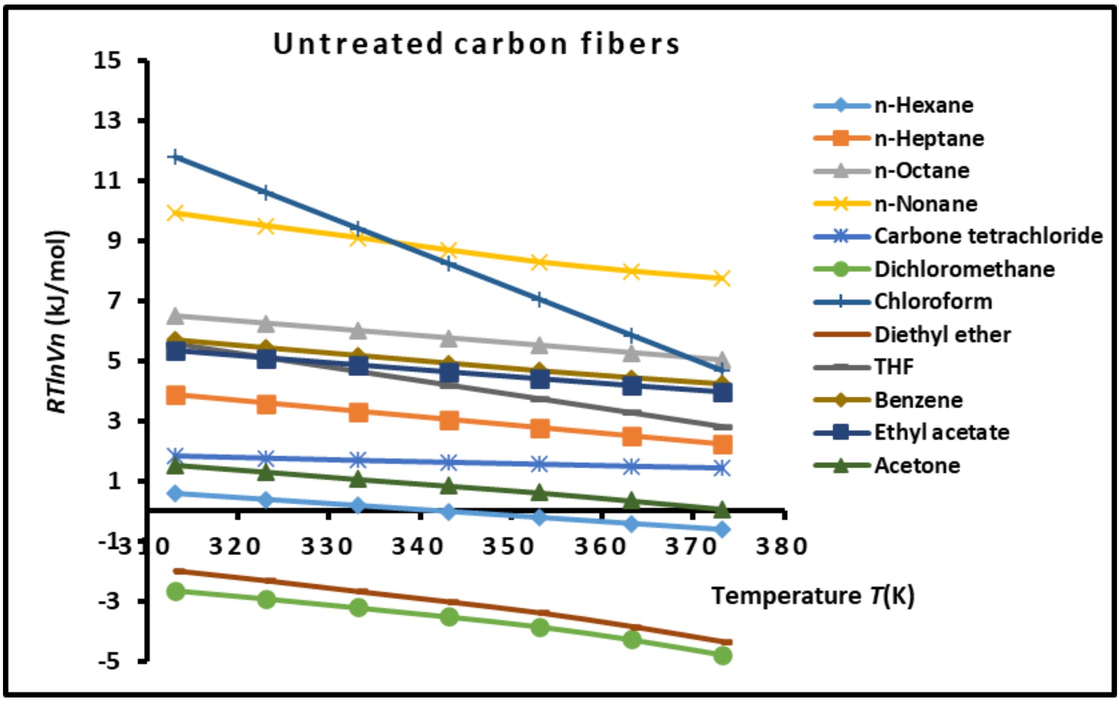

(d)

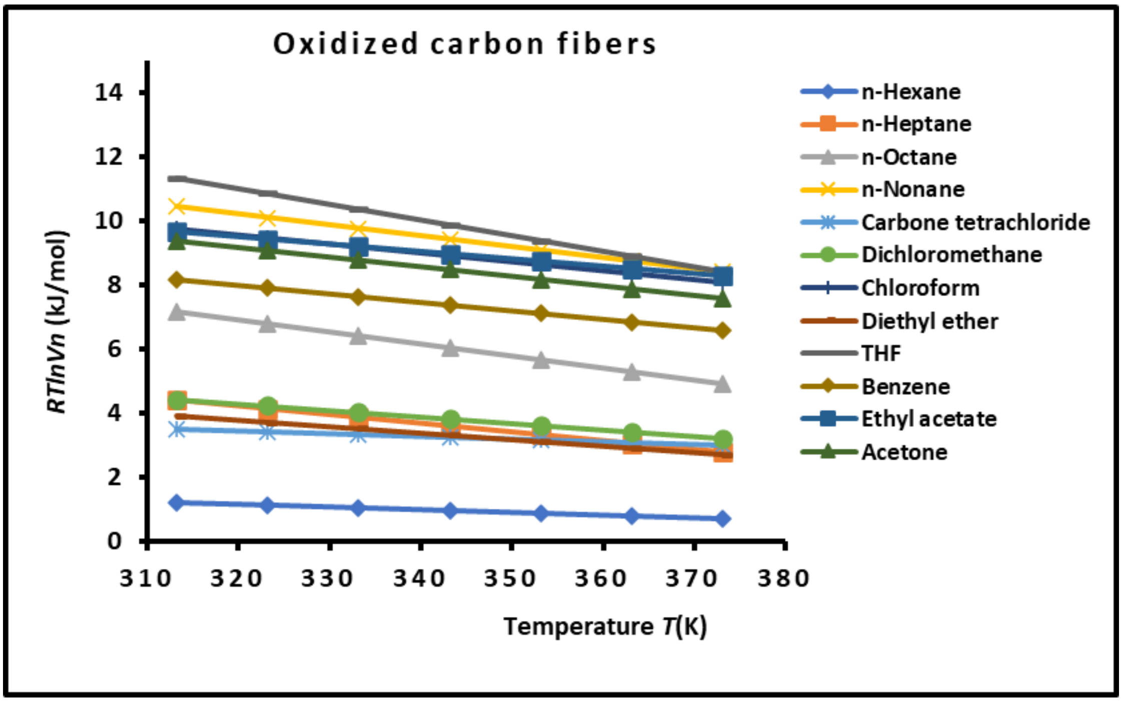

(e)

Figure S1. Variations of $RTlnV_n(T)$ of n-alkanes and polar molecules adsorbed on the various solid materials against the temperature. (a): Graphene, (b): Graphene oxide, (c): Reduced graphene oxide, (d) Untreated carbon fibers, and (e): Oxidized carbon fibers.

Table S1. Values of $-\Delta G_a^p(T)$ (in kJ/mol) of polar molecules adsorbed on the various solid materials against the temperature. Graphene, Graphene oxide, Reduced graphene oxide, Untreated carbon fibers, and Oxidized carbon fibers.

$-\Delta G_a^p(T)$ (in kJ/mol)			Graphene			
T(K)	Dichloromethane	Diethyl ether	THF	Ethyl acetate	Acetone	Acetonitrile
313.15	12.938	21.537	20.104	22.191	25.247	32.890
323.15	12.993	20.456	19.156	21.658	24.461	33.199
333.15	13.048	19.376	18.207	21.125	23.676	33.508
343.15	13.102	18.294	17.258	20.591	22.889	33.816
353.15	13.157	17.214	16.310	20.057	22.104	34.125
363.15	13.211	16.134	15.362	19.524	21.318	34.434
373.15	13.265	15.052	14.412	18.990	20.531	34.742
$-\Delta G_a^p(T)$ (in kJ/mol)			Graphene oxide			
T(K)	Dichloromethane	Diethyl ether	THF	Ethyl acetate	Acetone	Acetonitrile
313.15	2.309	9.736	8.668	11.657	12.238	18.469
323.15	2.749	9.445	8.404	11.603	12.414	19.019
333.15	3.192	9.157	8.143	11.552	12.592	19.571
343.15	3.639	8.873	7.887	11.504	12.772	20.122
353.15	4.082	8.584	7.626	11.452	12.950	20.674
363.15	4.523	8.294	7.363	11.400	13.127	21.225
373.15	4.964	8.003	7.100	11.347	13.304	21.775
$-\Delta G_a^p(T)$ (in kJ/mol)			Reduced graphene oxide			
T(K)	Dichloromethane	Diethyl ether	THF	Ethyl acetate	Acetone	Acetonitrile
313.15	10.175	20.953	17.327	21.696	20.831	19.905
323.15	10.148	19.968	16.364	20.849	20.987	20.995
333.15	10.123	18.983	15.403	20.000	21.141	22.080
343.15	10.095	17.995	14.438	19.151	21.296	23.168
353.15	10.068	17.009	13.474	18.302	21.451	24.257
363.15	10.042	16.023	12.512	17.453	21.604	25.341
373.15	10.013	15.035	11.547	16.604	21.758	26.429
$-\Delta G_a^p(T)$ (in kJ/mol)			Untreated carbon fibers			
T(K)	Dichloromethane	Diethyl ether	THF	Ethyl acetate	Acetone	Chloroform
313.15	4.130	2.133	11.809	9.471	10.681	15.339
323.15	3.872	1.873	11.455	9.381	10.499	14.255
333.15	3.614	1.613	11.101	9.291	10.317	13.171
343.15	3.356	1.353	10.747	9.201	10.135	12.087
353.15	3.098	1.093	10.393	9.111	9.953	11.003
363.15	2.840	0.833	10.039	9.021	9.771	9.919
373.15	2.582	0.573	9.685	8.931	9.589	8.835
$-\Delta G_a^p(T)$ (in kJ/mol)			Oxidized carbon fibers			
T(K)	Dichloromethane	Diethyl ether	THF	Ethyl acetate	Acetone	Chloroform
313.15	10.526	7.399	17.009	13.204	17.919	12.794
323.15	10.235	7.187	16.443	12.962	17.462	12.519
333.15	9.944	6.975	15.877	12.720	17.005	12.244
343.15	9.653	6.763	15.311	12.478	16.548	11.969

353.15	9.362	6.551	14.745	12.236	16.091	11.694
363.15	9.071	6.339	14.179	11.994	15.634	11.419
373.15	8.780	6.127	13.613	11.752	15.177	11.144

Table S2. Values of ($-\Delta H_a^p$ in kJ mol^{-1}) of polar molecules adsorbed on the different graphene and carbon materials

Materials	Dichloromethane	Diethyl ether	THF	Ethyl acetate	Acetone	Acetonitrile
Graphene	11.234	55.379	49.808	38.894	49.855	23.224
Graphene oxide	-11.565	18.765	16.833	13.261	6.6652	1.2085
Reduced graphene oxide	11.017	51.84	47.496	48.277	21.295	23.168
Untreated carbon fibers	12.209	49.284	10.275	22.895	12.289	-
Oxidized carbon fibers	19.639	21.406	14.038	34.733	20.782	-

Table S3. Values of ($-\Delta S_a^p$ in J K^{-1}mol^{-1}) of polar molecules adsorbed on the different graphene and carbon materials

Materials	Dichloromethane	Diethyl ether	THF	Ethyl acetate	Acetone	Acetonitrile
Graphene	-5.4	-44.3	2.7	25.8	29.1	-5.4
Graphene oxide	108.1	28.8	98.6	108.4	27.5	108.1
Reduced graphene oxide	94.9	26.1	96.3	26.0	21.2	94.9
Untreated carbon fibers	53.3	5.1	84.9	35.4	56.6	-
Oxidized carbon fibers	78.6	-17.8	-15.4	9.0	24.2	-

Table S4. Values of polar acid-base energies γ_s^+, γ_s^-, γ_s^p, and total surface energy $\gamma_s^{tot.}$ (in mJ/m^2) of the different graphenes and carbon materials at various temperatures.

Materials	T(K)	313.15	323.15	333.15	343.15	353.15	363.15	373.15
Graphene	γ_s^-	167.08	162.07	157.32	152.79	148.51	144.41	140.52
	γ_s^+	73.55	68.61	63.95	59.53	55.36	51.42	47.70
	γ_s^p	221.71	210.91	200.61	190.75	181.34	172.35	163.73
	$\gamma_s^{tot.}$	500.90	472.02	443.90	416.53	389.88	363.98	338.77
Graphene oxide	γ_s^-	5.32	7.25	9.41	11.79	14.29	16.93	19.68
	γ_s^+	20.29	19.69	19.12	18.58	18.05	17.53	17.03
	γ_s^p	20.78	23.91	26.84	29.60	32.12	34.45	36.61
	$\gamma_s^{tot.}$	139.0	133.2	127.4	121.7	116.1	110.5	105.0
Reduced graphene oxide	γ_s^-	103.34	98.87	94.69	90.70	86.96	83.44	80.07
	γ_s^+	70.30	63.58	57.32	51.50	46.10	41.09	36.46
	γ_s^p	170.47	158.57	147.35	136.69	126.62	117.11	108.06
	$\gamma_s^{tot.}$	321.4	305.8	290.7	275.9	261.6	247.4	233.5
Untreated carbon fibers	γ_s^-	17.03	14.39	12.07	10.02	8.23	6.67	5.32
	γ_s^+	13.40	12.87	12.37	11.89	11.42	10.98	10.55
	γ_s^p	30.20	27.22	24.44	21.83	19.40	17.12	14.99

	$\gamma_s^{tot.}$	86.5	80.2	74.4	68.5	62.9	58.2	53.9
Oxide carbon fibers	γ_s^-	110.59	100.57	91.37	82.93	75.19	68.08	61.56
	γ_s^+	26.04	24.58	23.19	21.86	20.60	19.41	18.27
	γ_s^p	107.33	99.43	92.06	85.16	78.72	72.70	67.07
	$\gamma_s^{tot.}$	163.2	151.0	139.4	128.4	117.9	107.9	98.4

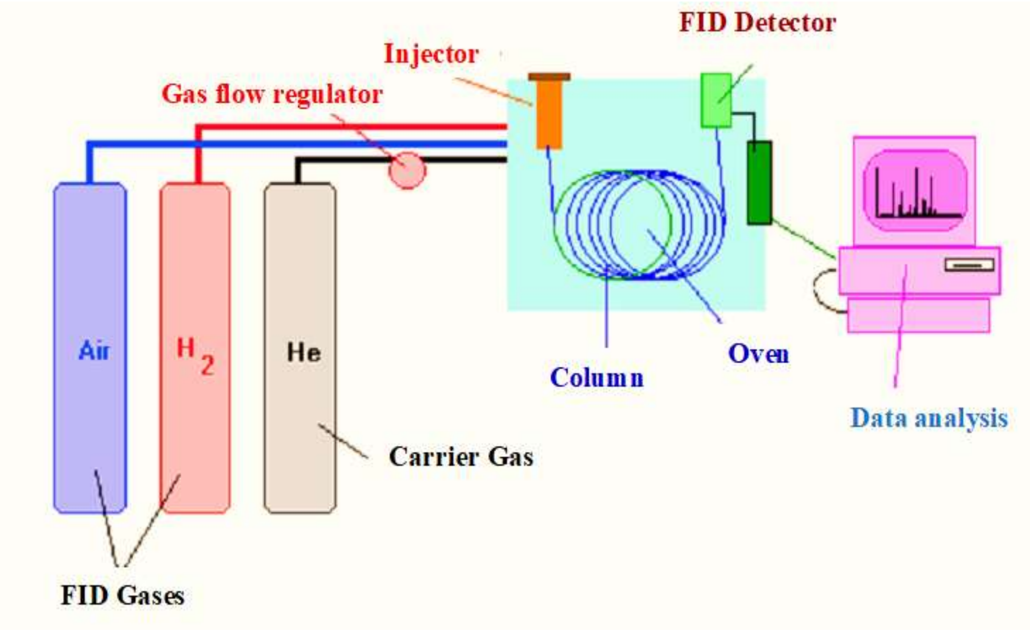

Figure S2. Schematic diagram of a gas chromatograph.
